# Association of the Triglyceride–Glucose Index During the First Trimester of Pregnancy with Adverse Perinatal Outcomes

**DOI:** 10.3390/diagnostics15091129

**Published:** 2025-04-29

**Authors:** Guillermo Gurza, Nayeli Martínez-Cruz, Ileana Lizano-Jubert, Lidia Arce-Sánchez, Blanca Vianey Suárez-Rico, Guadalupe Estrada-Gutierrez, Araceli Montoya-Estrada, José Romo-Yañez, Juan Mario Solis-Paredes, Johnatan Torres-Torres, Isabel González-Ludlow, Ameyalli Mariana Rodríguez-Cano, Maricruz Tolentino-Dolores, Otilia Perichart-Perera, Enrique Reyes-Muñoz

**Affiliations:** 1 Coordination of Endocrinology, Instituto Nacional de Perinatología Isidro Espinosa de los Reyes, Mexico City 11000, Mexico; gurzaguillermo@gmail.com (G.G.); li_arce@yahoo.com.mx (L.A.-S.); 2 Coordination of Gynecological and Perinatal Endocrinology, Instituto Nacional de Perinatología Isidro Espinosa de los Reyes, Mexico City 11000, Mexico; ileana1500@gmail.com (I.L.-J.); blancasuarezrico@gmail.com (B.V.S.-R.); jryz@yahoo.com (J.R.-Y.); 3 Facultad de Ciencias de la Salud, Universidad Anáhuac México, Campus Norte, Huixquilucan 52786, Mexico; 4 Department of Immunobiochemistry, Instituto Nacional de Perinatología Isidro Espinosa de los Reyes, Mexico City 11000, Mexico; gpestrad@gmail.com; 5 Clinical Research Branch, Instituto Nacional de Perinatología Isidro Espinosa de los Reyes, Mexico City 11000, Mexico; juan.mario.sp@gmail.com (J.M.S.-P.); johnatan.torres@inper.gob.mx (J.T.-T.); 6 Department of Nutrition and Bioprogramming, Instituto Nacional de Perinatología Isidro Espinosa de los Reyes, Mexico City 11000, Mexico; isaglezludlow@icloud.com (I.G.-L.); rocameyalli@gmail.com (A.M.R.-C.); cruz_tolentino@yahoo.com.mx (M.T.-D.); oti_perichart@yahoo.com (O.P.-P.)

**Keywords:** triglyceride–glucose index, perinatal outcomes, gestational diabetes, preeclampsia, pregnancy

## Abstract

**Background/Objectives**: Insulin resistance during pregnancy is a key factor underlying gestational diabetes mellitus (GDM) and other adverse perinatal outcomes (APOs). While traditional markers, such as HOMA-IR, are used to evaluate insulin resistance, they may be inaccessible in resource-limited settings. The triglyceride–glucose (TyG) index has emerged as a practical alternative. This study aimed to assess whether or not a TyG index > 8.6 during the first trimester of pregnancy is associated with an increased risk of APOs, including GDM, preeclampsia, and other maternal and neonatal complications. **Methods**: A prospective cohort study was conducted involving 333 pregnant women in Mexico City, divided into two groups: Group 1 (TyG index > 8.6, *n* = 153) and Group 2 (TyG index ≤ 8.6, *n* = 180). Primary outcomes included gestational diabetes mellitus (GDM), hypertensive disorders of pregnancy, preeclampsia, preterm birth, cesarean section, and large-for-gestational-age (LGA) and small-for-gestational-age (SGA) neonates. Logistic regression models were used to calculate the adjusted relative risk (aRR) and 95% confidence intervals (CIs), adjusting for maternal age, pregestational weight, and body mass index (BMI). **Results**: Women with a TyG index > 8.6 had a significantly higher pregestational weight and BMI than those with a TyG index ≤ 8.6. Group 1 demonstrated a higher risk of GDM (RR 2.05; 95% CI: 1.23–3.41) and preeclampsia (RR 2.15; 95% CI: 1.10–4.21). After adjusting for maternal age, pregestational weight, and BMI, these associations remained significant: GDM (aRR 1.87; 95% CI: 1.0–2.5) and preeclampsia (aRR 2.18; 95% CI: 1.1–5.0). No significant associations were found between an elevated TyG index and other APOs, including LGA, SGA, preterm birth, or cesarean delivery. **Conclusions**: A first-trimester TyG index > 8.6 is significantly associated with an increased risk of GDM and preeclampsia, highlighting its potential as a predictive marker for adverse perinatal outcomes. These findings underscore the utility of the TyG index as a practical, cost-effective tool for early risk stratification, particularly in resource-limited settings. Further multi-center research is needed to validate these results and refine population-specific thresholds.

## 1. Introduction

Pregnancy is a notable and complex physiological process characterized by dynamic metabolic changes designed to support fetal development [1,2]. As gestation progresses, nutritional demands increase, peaking during the third trimester [3]. Insulin plays a central role in glucose metabolism, facilitating its utilization as an energy source [4]. However, insulin sensitivity is sometimes impaired, leading to pathological metabolic conditions that can negatively affect perinatal outcomes [3,5]. The American Diabetes Association (ADA) defines insulin resistance as a condition characterized by decreased tissue responsiveness to insulin [6]; on the other hand, gestational diabetes mellitus (GDM) is defined as a carbohydrate intolerance that develops during pregnancy [7]. According to the Atlas of the International Diabetes Federation, an estimated 21.1 million (16.7%) live births in 2021 were affected by some form of diabetes during pregnancy, with 80.3% attributed to GDM [8].

Various indices have been used in clinical practice to assess insulin resistance, including the Homeostasis Model Assessment for Insulin Resistance (HOMA-IR) and the Quantitative Insulin Sensitivity Check Index (QUICKI) [9]. Both have been shown to predict GDM in the first trimester [10]. However, these methods require specific laboratory measurements that may not be readily accessible in resource-limited settings. In this context, the triglyceride–glucose (TyG) index has emerged as a promising marker due to its simplicity and efficacy, particularly during the first trimester of pregnancy [10]. This index, calculated using fasting triglyceride and glucose levels, has proven useful for predicting GDM and identifying altered fetal growth patterns [10].

Recent evidence highlights the predictive capability of the TyG index in identifying women at elevated risk of GDM and large-for-gestational-age (LGA) fetuses [11,12]. This marker allows for early intervention and closer monitoring in pregnancies prone to metabolic complications [13]. While previous studies have documented its association with GDM and LGA, critical gaps remain in the literature regarding its relationship with a broader spectrum of adverse perinatal outcomes (APOs) [10,11,12,13,14,15,16,17].

Beyond its association with metabolic indicators, recent advances in understanding the cellular and molecular mechanisms underlying GDM provide valuable insights into its pathophysiology and implications for maternal and fetal health. As highlighted in recent research, GDM is characterized by mitochondrial dysfunction, disruptions in insulin signaling, epigenetic alterations, and chronic inflammation, all of which contribute to insulin resistance and impaired glucose metabolism [18,19]. Elevated levels of reactive oxygen species (ROS) and inflammatory cytokines, such as TNF-α and IL-6, exacerbate these processes, creating a hostile intrauterine environment that affects placental function and fetal development [18]. The interplay of these mechanisms underscores the importance of identifying early, accessible biomarkers like the TyG index to predict adverse outcomes and mitigate long-term complications. By capturing the metabolic and inflammatory dysregulation seen in GDM, this index offers a practical tool for early risk assessment, particularly in resource-limited settings. Incorporating these insights strengthens the foundation for exploring novel preventive and therapeutic strategies.

Additionally, studies suggest that an elevated TyG index may be linked to hypertensive disorders of pregnancy, such as preeclampsia, further expanding its potential clinical utility [17]. 

Other potential APOs include preterm birth, neonatal intensive care unit (NICU) admissions, and small-for-gestational-age (SGA) infants. However, these findings are limited and often inconsistent.

Given the paucity of research on the utility of the TyG index in Latin American populations, this area presents a critical opportunity to enhance our understanding of its implications in different ethnic and demographic contexts. This study aimed to assess the association between a TyG index > 8.6, measured during the first trimester of pregnancy, and the risk of adverse perinatal outcomes, explicitly focusing on GDM and preeclampsia.

## 2. Materials and Methods

### 2.1. Study Design and Participants

This prospective cohort design study involved pregnant women enrolled in the “Biochemical and Epigenetic Origins of Overweight and Obesity” (OBESO) cohort. The protocol was reviewed and approved by the research, ethics, and biosafety committees of the National Institute of Perinatology (INPer) under registration number 3300-11402-01-575-17. Informed consent was obtained from all participants before enrollment. The study included women receiving prenatal care at the INPer from the first trimester of pregnancy until delivery, from 2017 to 2019. The inclusion criteria were a singleton pregnancy before 15 weeks of gestation, a maternal age >18 years, prenatal care, and pregnancy resolution in the INPer. Exclusion criteria included multiple pregnancies, pregestational diabetes mellitus (confirmed through clinical history, fasting glucose, and glycated hemoglobin levels measured during the first trimester), or the presence of any of the following conditions: hyperthyroidism, systemic lupus erythematosus, rheumatoid arthritis, heart disease, chronic hypertension, kidney disease, or liver disease. Participants were also excluded if they missed two or more scheduled prenatal visits or opted to withdraw from the study.

### 2.2. Data Collection

Participants were recruited during their first-trimester ultrasound appointments between 11.0 and 13.6 weeks of gestation. Blood samples were collected from individuals who met the inclusion criteria. These samples were processed immediately by centrifuging at 5000 rpm for 10 min and stored at −70 °C to ensure preservation until further analysis. Fasting glucose and triglyceride levels were measured in serum samples obtained after 8 to 10 h of fasting. Both parameters were analyzed using a colorimetric enzymatic assay at 540 nanometers, performed on the respons^®^910 analyzer (DiaSys Diagnostic Systems GMBH, Holsheim, Germany). The assay demonstrated a sensitivity of 20 mg/dL, with a coefficient of variation below 5%, ensuring the high reliability and reproducibility of the measurements.

### 2.3. Study Variables

The exposure variable in this study was insulin resistance, defined by a TyG index > 8.6, based on the value of the 75th percentile of a group of 96 Mexican women who were between 11 and 14 weeks of gestation, between 18 and 35 years old, and had a pregestational body mass index (BMI) of 18.5–24.9 kg/m^2^, with a normal 2-h, 75-g oral glucose tolerance test and exclusion of any co-morbidities, which were evaluated previous to the present study. The percentiles of 25, 50, and 75 were 8.0, 8.3, and 8.6, respectively, and represent cutoffs similar to previous research conducted in Latin American women [20]. The percentiles 25, 50, and 75 for TG were 92, 119, and 152 mg/dL, respectively. The TyG index was calculated using the following formula [21]: Ln (TG [mg/dL] × glucose [mg/dL]/2). Participants were divided into two groups: Group 1 included women with a TyG index > 8.6, while Group 2 consisted of those with a TyG index ≤ 8.6. The primary outcome variables assessed in this study were GDM, gestational hypertension, preeclampsia, preterm birth, LGA, and SGA. GDM was defined as one or more abnormal values during the 2-h, 75-g oral glucose tolerance test (OGTT), following the criteria established by the International Association of Diabetes and Pregnancy Study Groups (IADPSG). Abnormal thresholds for GDM included fasting glucose levels ≥ 92 mg/dL, glucose levels ≥ 180 mg/dL at 1 h, and glucose levels ≥ 153 mg/dL at 2 h after the glucose load [22]. Gestational hypertension was defined as a systolic blood pressure ≥ 140 mmHg and/or a diastolic blood pressure ≥ 90 mmHg in a previously normotensive woman after 20 weeks of gestation, without the presence of proteinuria or severity criteria [23]. Preeclampsia was defined as systolic blood pressure ≥ 140 mmHg and/or diastolic blood pressure ≥ 90 mmHg in a previously normotensive woman after 20 weeks of gestation, accompanied by proteinuria and/or severity criteria [23]. Preterm birth was defined as any delivery occurring between 20 and 36.6 weeks of gestation [24]. Neonates were classified as LGA if their weight was at or above the 90th percentile and as SGA if their weight was at or below the 10th percentile, based on gestational age and sex-specific birth weight references for the Mexican population [25].

### 2.4. Sample Size

The sample size calculation was based on detecting a 15% difference in the incidence of GDM between women with a TyG index > 8.6 and those with a TyG index ≤ 8.6. This calculation was performed with a significance level (alpha) of 0.05 and a statistical power of 80%. Based on these parameters, it was determined that a minimum of 100 participants were required for each group to ensure adequate statistical reliability.

### 2.5. Statistical Analysis

Continuous variables were described as means with standard deviations, while categorical variables were summarized as frequencies and proportions. Depending on the distribution of the data, comparisons of continuous variables were performed using either Student’s *t*-test or the Mann–Whitney U test. Differences in proportions were assessed using the chi-square test or Fisher’s exact test, as appropriate. Statistical significance was defined as a *p*-value ≤ 0.05. The relative risk (RR), with corresponding 95% confidence intervals (CIs), was calculated for each adverse perinatal outcome to evaluate associations. Logistic regression models were constructed for each outcome to account for potential confounding variables (i.e., variables with significant statistical differences between groups in a bivariate analysis). All statistical analyses were conducted using SPSS software, version 24 (Chicago, IL, USA).

## 3. Results

### 3.1. Baseline Characteristics

**Figure 1:**
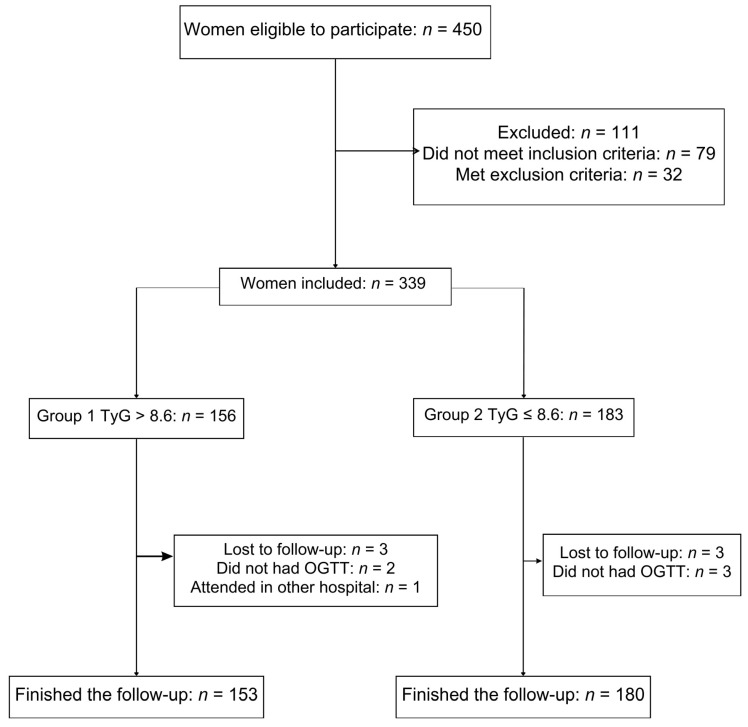
Cohort flow diagram of patient inclusion in the study.

During the study period, 450 pregnant women were eligible; of them, 339 women met the inclusion criteria, and 333 women finished the follow-up: Group 1 (*n* = 153) and Group 2 (*n* = 180) and were analyzed (Figure 1).

The baseline characteristics of the participants at the time of entry to prenatal care are shown in Table 1. No significant differences were observed between the groups regarding height, nulliparity, and the number of previous gestations. However, maternal age, pregestational weight, and pregestational BMI were significantly higher in women with a TyG index > 8.6 compared to those with a TyG index ≤ 8.6 (*p* = 0.01 for all). As anticipated, fasting glucose, triglyceride levels, and glycated hemoglobin (HbA1c) were also notably elevated in Group 1 (*p* < 0.0001 for all), reflecting the metabolic differences associated with an altered TyG index.

### 3.2. Characteristics at the Time of OGTT and Pregnancy Resolution

The maternal characteristics at the gestational age when the OGTT was performed are summarized in Table 2. Significant differences were observed in all three glucose measurements of the OGTT. Women in Group 1 (TyG index > 8.6) exhibited higher fasting glucose levels, as well as elevated glucose levels at 1 h and 2 h post-glucose-load, with all differences achieving statistical significance (*p* < 0.0001). Regarding pregnancy outcomes, no significant differences were identified between the groups regarding aspirin or metformin use during pregnancy, gestational weight gain, gestational age at delivery, or newborn birth weight. Metformin was indicated as part of the pharmacologic management of GDM. These findings suggest that other clinical factors and perinatal outcomes remained comparable despite the metabolic differences between the groups.

### 3.3. Risk of APOs in Both Groups

The analysis of APOs is summarized in Table 3. Women with a TyG index > 8.6 demonstrated a significantly higher risk of developing GDM and preeclampsia. Specifically, the RR for GDM was 2.05 (95% CI: 1.23–3.41), while the RR for preeclampsia was 2.15 (95% CI: 1.10–4.21). These associations remained significant after adjusting for confounding variables, with adjusted relative risks (aRR) of 1.87 (95% CI: 1.0–2.5) for GDM and 2.38 (95% CI: 1.1–5.0) for preeclampsia.

In terms of fetal weight, no statistically significant associations were observed for SGA neonates (RR 1.031; 95% CI: 0.96–1.101) or LGA neonates (RR 2.18; 95% CI: 0.89–5.33). Similarly, there was no increased risk for other APOs, including gestational hypertension, preterm birth, or cesarean delivery. These findings highlight the association between an elevated TyG index and the risk of GDM and preeclampsia, while other outcomes appeared unaffected by maternal metabolic status.

A logistic regression analysis was conducted to adjust for potential confounding variables, including maternal age, pregestational weight, gestational weight gain, BMI, and the TyG index. The results indicated that a high TyG index during the first trimester (TyG > 8.6) was significantly associated with an increased risk of gestational diabetes mellitus (aRR 1.87; 95% CI: 1.0–2.5) and preeclampsia (aRR 2.18; 95% CI: 1.1–5.0), independent of maternal age, pregestational weight, and BMI. However, no significant associations were observed for gestational hypertension, LGA neonates, SGA neonates, preterm birth, vaginal delivery, or cesarean section.

## 4. Discussion

### 4.1. Principal Findings

This study demonstrated that Mexican pregnant women with a TyG index > 8.6 during the first trimester have a significantly increased risk of developing GDM and preeclampsia. These findings emphasize the potential of the TyG index as an accessible and reliable biomarker for identifying high-risk pregnancies early in gestation. Notably, no associations were found between an elevated TyG index and other APOs, such as LGA or preterm birth, which suggests a more specific relationship between metabolic status and the identified complications.

### 4.2. Comparison with Existing Literature

Our results regarding GDM align with previous studies in other populations. Song et al. conducted a systematic review and meta-analysis that reported a significant association between an elevated TyG index and an increased risk of GDM, with an odds ratio (OR) ranging from 2.04 to 3.30 (*p* < 0.05) [14].

Similarly, Guo et al. demonstrated in a large prospective cohort of 1624 Chinese pregnant women that a first-trimester TyG index > 8.89 predicted GDM, highlighting the index’s ability to stratify metabolic risk early in pregnancy [13]. While Li et al. suggested a slightly lower cutoff value of 8.55 for Chinese women [12], the variability in thresholds across studies underscores the need to establish population-specific reference values, particularly for Latin American women. The lack of a universally accepted cutoff further complicates the direct comparison of findings, as some authors, such as Ye et al., have combined the TyG index with other biomarkers, like HbA1c, to enhance predictive accuracy [26].

Limited research has explored the association between preeclampsia and the TyG index. Our findings suggest that a TyG index > 8.6 is a significant risk factor for preeclampsia, consistent with Ye et al.’s study, which reported a similar association using a higher threshold (>8.98) [26]. These findings are particularly relevant, as they expand the clinical utility of the TyG index beyond GDM to hypertensive disorders of pregnancy, offering new opportunities for early identification and targeted interventions.

In contrast, no significant relationship between a TyG index > 8.6 and LGA neonates or preterm birth was observed. This finding is inconsistent with the findings of Lin et al., who reported an increased risk of LGA when the TyG index exceeded 8.23 [15], and Zhang et al., who found modest predictive performance for preterm birth and macrosomia in a cohort of 8514 participants [27]. These discrepancies may be attributed to differences in study populations, sample sizes, incidence of APOs, and methodological approaches. Additionally, the influence of genetic, environmental, and nutritional factors specific to Latin American populations could play a role in modulating these associations, further highlighting the need for regional studies to validate these findings.

An important aspect that reinforces the findings of our study is the practicality of the TyG index as a marker of insulin resistance compared to more complex methods, such as HOMA-IR. According to Tahapary et al., the TyG index offers a more accessible alternative for resource-limited populations, as it only requires fasting triglyceride and glucose measurements, eliminating the need for insulin assays, which are more costly and difficult to implement in low-resource clinical settings [28]. Furthermore, this index has demonstrated a strong correlation with the hyperinsulinemic–euglycemic clamp, the gold standard for evaluating insulin resistance [28].

### 4.3. Strengths and Limitations

The strengths of this study include its robust sample size, which represents the largest cohort of pregnant Latin American women analyzed to date using the TyG index. The prospective design and consistent follow-up during prenatal care enhance the reliability of the findings. Additionally, all laboratory analyses were conducted in a single center, ensuring standardization of data collection and minimizing inter-laboratory variability.

However, in this study, several limitations must be acknowledged. As a single-center study, the findings may not be generalizable to other healthcare settings or populations. Furthermore, we lacked detailed data on potentially confounding variables, such as dietary habits, sleep patterns, and family histories of metabolic or hypertensive disorders. The absence of these factors limits our ability to fully account for their influence on the observed associations. Additionally, while the TyG index is a simple and cost-effective marker, its utility could be enhanced by exploring combinations with other biomarkers or clinical parameters to improve predictive accuracy.

### 4.4. Clinical Implications

The clinical implications of these findings are significant. The TyG index offers a practical, inexpensive, and easily accessible tool for early risk stratification in pregnancy, particularly in low- and middle-income settings where more complex measures of insulin resistance, such as HOMA-IR, are often impractical [28]. The main pathophysiological process causing hyperglycemia in GDM is a failure of insulin secretion, or relative insulin deficiency, to compensate for insulin resistance, which might be pre-existing, in addition to the insulin resistance elevation during mid to late pregnancy [2]. The most common pathophysiological subtype of GDM is insulin resistant, typically accounting for 50–60% of GDM cases. Insulin resistance and defects in insulin secretion present before pregnancy or in early pregnancy [2]. The TyG index has emerged as a reliable surrogate marker for identifying insulin resistance and metabolic disorders due to its simplicity and practicality [13]. Although physiological insulin resistance during pregnancy is beneficial for fetal growth and nutrient supply, if the degree of insulin resistance is significantly higher than that of normal pregnancy, it may lead to abnormal glucose metabolism, chronic inflammation, and oxidative stress, thereby creating an in utero environment enriched with glucose, lipids, amino acids, and other nutrients, leading to GDM, preeclampsia, intrauterine growth restriction, and fetal macrosomia, among other complications [10].

By identifying women at a higher risk of GDM and preeclampsia during the first trimester, healthcare providers can implement targeted interventions, such as nutritional counseling, pharmacological therapies, or enhanced monitoring, to mitigate these risks. Moreover, incorporating the TyG index into routine prenatal screening protocols could enhance the identification of high-risk pregnancies and improve maternal and neonatal outcomes. Future research should focus on validating these findings in larger, multi-center studies and exploring the integration of the TyG index into predictive models that account for additional clinical and metabolic factors.

### 4.5. Research Implications

The findings of this study open several avenues for future research on the role of the TyG index in maternal-fetal medicine. First, there is a critical need to conduct large-scale, multi-center studies to validate the observed associations in diverse populations, particularly in underrepresented regions such as Latin America. These studies aim to refine the optimal cutoff values for the TyG index to enhance its predictive accuracy for GDM, preeclampsia, and other APOs. Furthermore, longitudinal studies investigating the trajectory of the TyG index throughout pregnancy could provide valuable insights into its dynamic relationship with evolving metabolic and inflammatory changes. Integrating advanced molecular techniques to explore the mechanistic pathways linking the TyG index to placental function, oxidative stress, and inflammation would also deepen our understanding of its role in pregnancy complications. Lastly, the potential for combining the TyG index with emerging biomarkers, such as adipokines or pro-inflammatory cytokines, should be explored to develop more robust predictive models that account for the multifactorial nature of these conditions.

## 5. Conclusions

A TyG index > 8.6 during the first trimester was significantly associated with an increased risk of adverse perinatal outcomes, specifically GDM and preeclampsia. These findings underscore the potential of the TyG index as an accessible and practical biomarker for early identification of high-risk pregnancies. However, further research with larger, multi-center cohorts is necessary to validate these results and establish population-specific thresholds. Such studies would facilitate the development of targeted interventions aimed at reducing the incidence and burden of adverse perinatal outcomes, ultimately improving maternal and neonatal health outcomes.

## Figures and Tables

**Table 1:** Baseline characteristics of the pregnant women according to the study group.

Characteristic	Group 1, TyG > 8.6 *n* = 153	Group 2, TyG ≤ 8.6 *n* = 180	*p*-Value
Maternal age (years)	30.8 ± 5.1	28.9 ± 5.4	0.01
Pregestational weight (kg)	69.7 ± 13.1	66.0 ± 14.9	0.01
Height (m)	1.57 ± 0.06	1.57 ± 0.5	0.49
Pregestational BMI (kg/m^2^)	28.15 ± 4.6	26.6 ± 5.5	0.01
First-trimester BMI (kg/m^2^)	28.4 ± 4.5	26.7 ± 5.7	0.03
Nulliparous	86 (56.2%)	113 (62.8%)	0.26
One or more previous gestations	67 (43.8%)	67 (37.2%)	0.26
Glucose (mg/dL)	86.0 ± 11.4	73.6 ± 10.3	0.0001
Triglycerides (mg/dL)	179 ± 44	106.2 ± 24.6	0.0001
HbA1c	5.3 ± 0.4	5.0 ± 0.5	0.0001

TyG: Triglyceride–glucose index; BMI: body mass index; HbA1c: glycated hemoglobin. Values are expressed as a mean ± standard deviation or frequency (percentage).

**Table 2:** Characteristics at the time of the glucose tolerance test and pregnancy resolution.

Characteristic	Group 1, TyG > 8.6 *n* = 153	Group 2, TyG ≤ 8.6 *n* = 180	*p*-Value
Weeks of gestation at OGTT (weeks)	22.2 ± 6.1	21.5 ± 5.7	0.24
Fasting OGTT (mg/dL)	82 ± 9.6	77.7 ± 8.2	0.0001
1-h OGTT (mg/dL)	138.2 ± 39	118 ± 32.1	0.0001
2-h OGTT (mg/dL)	117.12 ± 28.5	103.3 ± 25.9	0.0001
Aspirin use	59 (38.5%)	53 (29.44%)	0.06
Metformin use	24 (15.7%)	18 (10%)	0.12
Gestational weight gain	6.6 ± 5.2	7.6 ± 5.3	0.16
Gestational age at resolution (weeks)	38.2 ± 1.7	38.3 ± 1.7	0.49
Newborn weight (g)	3019 ± 502	2987 ± 416	0.52

TyG: Triglyceride–glucose index; OGTT: oral glucose tolerance test.

**Table 3:** Risk of adverse perinatal outcomes in women with a TyG index > 8.6.

Perinatal Outcome	Group 1, TyG > 8.6 (*n* = 153)	Group 2, TyG ≤ 8.6 (*n* = 180)	RR (95% CI)	aRR(95% CI)	*p*-Value
LGA	11 (7.2%)	18 (10%)	1.03 (0.96–1.10)	2.10 (0.81–5.6)	0.36
SGA	13 (8.5%)	7 (3.9%)	2.18 (0.89–5.33)	0.70 (0.31–0.61)	0.07
Preterm birth	22 (14.4%)	19 (10.5%)	1.42 (0.79–2.55)	1.56 (0.75–3.22)	0.31
Gestational diabetes mellitus	35 (22.9%)	20 (11.1%)	2.05 (1.23–3.41)	1.87 (1.0–2.5)	0.004
Preeclampsia	22 (14.4%)	12 (6.7%)	2.15 (1.10–4.21)	2.38 (1.1–5.0)	0.021
Gestational hypertension	6 (3.9%)	5 (2.8%)	1.41 (0.43–4.53)	1.66 (0.44–6.18)	0.56
Vaginal delivery	65 (42.5%)	66 (36.7%)	1.12 (0.85–1.47)	1.3 (0.85–2.22)	0.26
Cesarean section	88 (57.5%)	115 (63.9%)	0.92 (0.78–1.10)	0.72 (0.44–1.7)	0.26

TyG: Triglyceride–glucose index; aRR: adjusted relative risk; LGA: large-for-gestational-age; SGA: small for gestational age. Maternal age, pregestational weight, and pregestational body mass index were adjusted for each perinatal outcome.

## Data Availability

The datasets generated and analyzed during the current study are not publicly available. However, anonymized data are available from the corresponding author upon reasonable request.
